# Mesothelium and Malignant Mesothelioma

**DOI:** 10.3390/jdb7020007

**Published:** 2019-04-08

**Authors:** Emilye Hiriart, Raymond Deepe, Andy Wessels

**Affiliations:** Department of Regenerative Medicine and Cell Biology, Medical University of South Carolina, 173 Ashley Avenue, Charleston, SC 29425, USA; hiriart.emilye@icloud.com (E.H.); deepe@musc.edu (R.D.)

**Keywords:** mesothelium, development, malignant, mesothelioma, cancer

## Abstract

The mesothelium is an epithelial structure derived from the embryonic mesoderm. It plays an important role in the development of a number of different organs, including the heart, lungs, and intestines. In this publication, we discuss aspects of the development of the mesothelium, where mesothelial structures can be found, and review molecular and cellular characteristics associated with the mesothelium. Furthermore, we discuss the involvement of the mesothelium in a number of disease conditions, in particular in the pathogenesis of mesotheliomas with an emphasis on malignant pleural mesothelioma (MPM)—a primary cancer developing in the pleural cavity.

## 1. Introduction

Malignant mesothelioma is a neoplasm that originates from mesothelial cells lining the body cavities, including the pleura, peritoneum, pericardium, and tunica vaginalis. The majority of malignant mesothelioma cases are mesotheliomas that develop in the pleural cavity. They are known as malignant pleural mesothelioma (MPM) and comprise 70–90% of all reported cases of malignant mesothelioma [1,2]. The other cases typically arise in the peritoneum [3], while the pericardium is rarely affected [4]. In this review we will briefly discuss the origin of the mesothelial structures, provide a succinct overview of molecular mechanisms involved in their development, and address aspects of the etiology and pathogenesis of mesotheliomas.

## 2. Developmental Origin of the Mesothelial Structures

The mesoderm is one of the three germ layers formed during early embryonic development. The mesoderm develops in the space between the two other germ layers, the ectoderm and the endoderm, in a process called gastrulation. Within the lateral plate mesoderm, we can distinguish two distinct layers. The dorsal layer is called the somatic mesoderm and is associated with the overlying ectoderm, while the ventral layer, the splanchnic mesoderm, is associated with the underlying endoderm. As the embryo continues to develop, coelomic cavities emerge and three more or less separate parts can be identified. The pericardial cavity houses the developing heart, the peritoneal cavity contains the developing viscera of the digestive system, and in the bilateral pleural cavities, the lungs will emerge. The mesoderm-derived coelomic epithelium lining the walls of these cavities is commonly referred to as the parietal mesothelium [5,6,7]. Finally, mesothelium is also found around the male and female reproductive organs [8,9]. During organogenesis, mesothelial derivatives subsequently cover the developing organs to form the visceral mesothelium, a process that varies from organ to organ [7]. The space found in between the parietal and visceral mesothelium is typically filled with a serosal fluid containing immunoglobulin, complement, lysozyme, and other proteins, collectively providing protection against bacterial infections and allowing the respective organs to move with a minimal amount of friction [9,10,11]. Despite the fact that mesothelium is found covering a variety of organs and lining a number of different body cavities, its structure and characteristics remain relatively consistent throughout the body [12,13,14]. In the adult, the mesothelium is a simple squamous epithelium found on the surface of all coelomic organs. At histological level, it is characterized by three features: apical/basolateral polarity, robust cell/cell adhesion, and the presence of a basement membrane [7]. For many years, the role of the mesothelium was considered limited to the organs contained in the coelomic cavities as a non-adhesive protective barrier enabling movement of coelomic organs against each other and the body wall. Indeed, the mesothelium has an important role in facilitating the peristalsis of the intestines and the rhythmic “swelling” of the lungs during breathing and beating of the heart. More recently, however, we have come to understand that the mesothelium plays a far more extensive and active biological role [15,16]. The three major organs that have an associated mesothelium are the lungs, heart, and the intestines.

### 2.1. The Pleural Mesothelium

In the pleural cavities, two populations of mesothelial cells can be distinguished based on their localization. The mesothelium covering the walls of the cavity is referred to as the parietal pleura while the mesothelium covering the surface of the lungs is called the visceral pleura. After undergoing mesothelial-to-mesenchymal transition (MMT), a process in which TGFβ1 plays an important role, a subset of (visceral) pleural mesothelial cells (PMCs) migrate into the parenchyma of the lungs where they differentiate into alpha smooth muscle-expressing bronchial smooth muscle cells, vascular smooth muscle cells, and fibroblasts [17,18,19]. Furthermore, in pathological conditions, such as idiopathic pulmonary fibrosis, PMCs can transition into myofibroblasts [20]. Several transcription factors, growth factors, and signaling pathways are involved in the development of the pleural mesothelium, including the transcription factors WT1, SOX2, as well as the Wnt/β-catenin and Hedgehog signaling pathways. In Section 3 we will discuss some of these molecular mechanisms in a little bit more detail. 

### 2.2. The Cardiac Mesothelium

The mesothelium covering the lining of the pericardial cavity is known as the (parietal) pericardium. The visceral mesothelium found on the surface of the heart is the epicardium. The first step in the formation of the epicardium is the development of the proepicardium, a cauliflower-shaped mesothelium-derived proliferation of cells located at the interface between liver and sinus venosus [21,22,23]. The epicardium forms in the looping stages of heart development when cells from the proepicardium attach to the myocardial surface at the developing atrioventricular junction [23,24,25]. From the area of initial attachment, the epicardial cells spread out over the surface of the heart, eventually covering the heart completely. An epicardial-to-mesenchymal transition (epiMT) of a subset of epicardial cells leads to the formation of epicardially-derived cells (EPDCs) [26] that move into the extracellular-rich space in between the epicardium and the underlying myocardium. From there, EPDCs migrate into the cardiac walls and cell fate studies have shown that within the myocardial walls; these EPDCs can differentiate into different cell types including interstitial fibroblasts, coronary smooth muscle, and coronary endothelial cells, although it needs to be mentioned that the contribution to the coronary endothelium is still a matter of debate and may differ between species [22,26,27,28,29,30,31]. Recent studies have shown that EPDCs are also very important in the establishment of the annulus fibrosus, which is the fibrous tissue responsible for separating and insulating the atrial and ventricular working myocardium, thereby preventing ventricular pre-excitation in the postnatal heart, and that they contribute to the mesenchyme of the developing atrioventricular valves where they eventually become valve interstitial cells (VICs) [31,32]. Insight into the molecular regulation of epiMT has largely been gathered from in vitro studies using chick and mouse epicardial explants and from the use of immortalized epicardial cell lines [33,34,35,36,37]. Combined, these studies have revealed a number of proteins involved in epiMT. This list includes the transcription factors WT1, Snail, and Slug; growth factors such as BMP2, TGFβ1 and TGFbeta2; and cell surface receptors, including PDGFRalpha, PDGFRbeta, TGFBR1/Alk5, TGFBR3, and BMPR1A/ALK3 [23,33,34,35,36,37,38,39,40].

### 2.3. The Serosal Mesothelium

Finally, the serosal mesothelium is the visceral layer of the peritoneum located on the walls of the gut. In the early stages of gut development, its primordium consists of endoderm, splanchnic mesoderm, a vascular plexus, and neural crest-derived cells, which eventually give rise to the neurons and glia of the enteric plexus [41]. At these stages, there are no cells in or on the gut that express markers commonly associated with the mesothelium, such as WT1 and cytokeratin [5,42]. Compared to the development of the epicardium on the surface of the heart, the development of the serosal mesothelium is a relatively late event [5,43]. For instance, whereas in the avian heart (quail, chick), the epicardium starts to form around day 3 of embryonic development [23], the definitive mesothelium on the gut of these embryos forms three days later [5,43]. Interestingly, the mechanism through which the serosal mesothelium becomes established appears to be quite different from what is seen in the development of the visceral pleura and the epicardium. In vitro studies using chick/quail explants suggest that mesothelial progenitors are broadly positioned and intrinsic to the gut primordium [5], a finding that was substantiated by other approaches, including retroviral cell fate tracing experiments. Despite these developmental differences the serosal mesothelium and the epicardium have a similar appearance [7]. 

## 3. Signaling Pathways and Transcriptional Control Involved in the Development of the Mesothelium

A series of studies over the years has shown that the mesothelial cells found in different organs often share the expression of specific characteristic transcription factors and use the same signaling mechanisms to interact with other cell types. Here we will briefly review some of them.

### 3.1. Wilms Tumor 1 (WT1)

The transcription factor *WT1* was first identified in 1990 as a tumor suppressor gene in renal nephroblastoma [44]. It is a zinc finger transcription factor expressed in visceral and parietal mesothelial cells, including those found in the peritoneal, pleural, and pericardial spaces [45], and in kidney podocytes and glomerular capillaries [46]. WT1 is necessary for the development of kidneys and gonads, where it controls cell growth, differentiation, and apoptosis [47]. In the heart, WT1 is involved in in regulation of epicardial retinoic acid (RA) signaling and in the regulation of proliferation, migration, and differentiation of epicardial cells and epicardially-derived cells (EPDCs) [48,49,50]. Loss of WT1 results in reduced cardiomyocyte mass, pericardial hemorrhage, thinning of the myocardial wall, and embryonic lethality [49,51]. Mutations in the *WT1* gene have also been associated with pulmonary dysplasia [52], hypoplastic lung malformation [53], diaphragmatic hernia [54], decreased mesothelial cell entry into the lung [18], and mesothelioma [55]. In addition, WT1 plays a crucial role in liver development. Mice that do not express WT1 show decreased levels of proliferation of hepatoblasts and exhibit premature differentiation of hepatic stellate cells leading to hypoplastic liver with defects in lobation [56]. Deletion of WT1 results in loss of hepatic mesothelial expression of the retinoic acid (RA) synthesizing enzyme, RALDH2 [57]. 

### 3.2. Retinoic Acid (RA) and Retinaldehyde Dehydrogenases (RALDHs)

RA is produced by retinaldehyde dehydrogenases (RALDH1, 2, and 3) [58]. The expression patterns of the respective RALDH isoforms differ during early embryogenesis, with RALDH2 being expressed in mesodermal and mesothelial cells [59]. RA, in combination with erythropoietin (EPO) produced by the liver, has been associated with epicardial-dependent myocardial proliferation, a process that involves insulin like growth factor-2 (IGF2) [60]. Secreted EPO binds to epicardial surface receptors after crossing the pericardial cavity [61]. In 2006, Desai and colleagues reported that RA is required for initial stages of embryogenesis and is essential for lungs development [62]. In the liver, the relationship between RA signaling and WT1 has been explored using the WT1 knockout mouse; loss of WT1 results in loss of hepatic mesothelial expression of RALDH2 [57]. Furthermore, molecular inhibition of RA synthesis in the chick was found to lead to a decrease in liver size, whereas treatment of hepatic explants with RA increased proliferation. These observations suggested that synthesis of RA by hepatic mesothelium is regulating hepatoblast proliferation [57].

### 3.3. Insulin Growth Factor 1 and 2 (IGF1 and IGF2)

IGF2 is expressed by the epicardium and has been identified as a mediator of cardiomyocyte proliferation [63]. Disruption of the expression of IGF2 or its receptors leads to decreased myocardial proliferation in mice [63]. Combined with data discussed above, it suggests that RA signaling, EPO, and IGF2 are involved in a molecular partnership governing the epicardial control of myocardial proliferation. In the lungs, IGF1 has been shown to be expressed by early postnatal pleural mesothelium and adult pleural cell lines [64,65]. However, the developmental expression and the function of IGF1 in the lungs remains unclear. 

### 3.4. Fibroblast Growth Factor (FGF) Signaling

Members of the FGF family have broad mitogenic and cell survival activity and are involved in many aspects of embryonic development. FGF9 is the most frequently studied family member in connection with the mesothelium. FGF9 is a 26 kDa secreted glycoprotein which has been detected in the mesothelial cells of several coelomic organs [66]. A number of studies have demonstrated the role of mesothelial FGF9 expression in the morphogenesis of coelomic organs. In 2005, Lavine and colleagues identified FGF9 as a potential downstream mediator of the mitogenic effects of RA [67]. Treatment of epicardial cells or explanted hearts with RA induced FGF9 expression in the epicardium and inhibition of FGF9 signaling in murine hearts led to decreased myocardial proliferation. FGF9 is, however, only expressed transiently in the epicardium in a brief period within a larger window during which the myocardium proliferates. This suggests that, in addition to FGF9 in the epicardium, other factors and/or other FGF9 expressing cell types are also involved in the regulation of myocardial proliferation [67]. In the lungs, both the mesothelium and the endodermal epithelium express FGF9. The FGF9 produced by the pulmonary mesothelium is thought to regulate mesenchymal proliferation during pulmonary development, while FGF9 secreted by the pulmonary endoderm regulates airway branching [68]. Mice that do not express FGF9 present with reduced airway branching and a decrease in mesenchymal cells, resulting in development of hypoplastic lungs [17]. Furthermore, FGF9 is also implicated in the negative control of visceral smooth muscle cell differentiation in the lungs. Specifically, it has been suggested that mesothelial FGF9 maintains the outer mesenchymal cells in an undifferentiated state [69]. FGF9 is also expressed in both the endoderm and the mesothelium of the intestines [67,70]. Mice deficient for FGF9 develop a shortened small intestine due to both decreased mesenchymal proliferation and premature differentiation. It is, however, not completely clear whether this is mainly the result of the absence of FGF9 from the endoderm or whether the absence of FGF9 from the mesothelial cells plays a role in the pathogenesis as well [71]. 

### 3.5. Hedgehog (Hh) Signaling

Hedgehog (HH) signaling is one of the critical signaling pathways controlling organogenesis throughout the entire embryo [72]. In the lungs, HH signaling regulates branching morphogenesis and mesenchymal cell proliferation and differentiation [73,74]. During lung development, sonic hedgehog (SHH) signaling regulates mesothelial cell migration into the lung parenchyma, where they undergo a transition to form subpopulations of bronchial smooth muscle cells, vascular smooth muscle cells, and fibroblasts [18,19,75]. This process has also been shown to occur in the adult [76,77,78]. SHH-null embryos display multiple organ defects, including hypoplastic lungs, impaired branching, and lack of smooth muscle cells in the lungs [79]. HH signaling in the serosal mesothelial cells is also critical for proper formation and function of the intestines [80]. 

The factors and pathways mentioned above are just a few associated with the development and function of the mesothelium. Other factors and pathways, including the BMPs, VEGF, and the Wnt/beta-catenin pathway, are found to be active in the mesothelial cell lineage and during the formation of coelomic organs as well. It is also important to note that epithelial-to-mesenchymal transformation (EMT) is a common characteristic of embryonic mesothelial structure.EMT is typically regulated by factors secreted by the tissues underneath. This process has been described, for instance, for the heart [32], intestines [81], lungs [75], and liver [57]. After undergoing EMT, the mesothelium-derived mesenchymal cells migrate in the underlying tissues where they subsequently differentiate into specialized cells that are essential for the further development of the respective organs [15]. 

## 4. Molecular Characteristics and Markers of the Mesothelium

The growing interest in the development of the mesothelium and the pathological conditions that involve mesothelium-derived structures has led to the identification and characterization of genes associated with these structures. In many cases, the expression of mesothelium-associated genes allows for the identification of mesothelial cells and the study of their behavior during development. The identification of mesothelial-specific genes has also allowed the generation of transgenic models to specifically trace and/or target genes and pathways important for the development and function of the mesothelium and mesothelium-derived cells and tissues [32,82,83]. Importantly, expression of characteristic mesothelium-associated genes can also be instrumental in the diagnosis of diseases involving the mesothelia that are associated with their respective organs. Below, we present, in random order, an overview of a number of genes and markers that have been identified as being relevant in the study of mesothelial structures and associated diseases. It is beyond the scope of this review to discuss all the markers and their usefulness in detail.

E-cadherin and CD44: E-cadherin expression is usually associated with epithelial tissues as a transmembrane glycoprotein that mediates calcium-dependent, homophilic cell–cell adhesion [84]. CD44 is a cell-surface glycoprotein involved in cell–cell interactions, cell adhesion, and migration. It is also a receptor for hyaluronic acid. CD44 along with β1 integrin heterodimers have been suggested to play a role in mediating the adhesion of ovarian carcinoma cells to mesothelial cells [85]. A study in which samples of pelvic washings from patients presenting a metastatic ovarian adenocarcinoma were compared with benign peritoneal washings, concluded that the combination of E-cadherin/CD44 is highly specific and a useful diagnostic tool to distinguish benign reactive mesothelial cells from adenocarcinoma [86].

ME1 and ME2: The monoclonal antibodies ME1 and ME2, which were initially generated by immunizing mice with the mesothelioma cell line SPC111 [87], have been used to study human cultured mesothelial cells and ovarian tumor cells. In combination with AUA1—an antibody which recognizes a human cell surface antigen on epithelial cells [88]—ME1 and ME2 recognized both normal mesothelial cells and mesothelioma. In the referenced paper, the authors concluded that AUA1 was more useful for positive identification of mesothelial cells [89]. 

Desmin, calretinin, and N-cadherin: N-cadherin is expressed in the mesothelium and can be used in combination with desmin to distinguish between reactive mesothelium and malignant epithelial cells [90]. Desmin can also be used in conjunction with calretinin, because calretinin is a sensitive marker for mesothelial cells in cytologic specimens [91]. It has also been described as a very useful marker for the positive identification of normal and tumor mesothelial cells in serous effusions [91].

Hyaluronan and phospholipids: Hyaluronan and phospholipids are known to be produced by mesothelial cells [92] and can be used as peritoneal fluid markers of mesothelial cells [93]. 

Mesothelin: Mesothelin is a cell surface glycoprotein and differentiation marker highly expressed in mesothelial cells lining the pleura, pericardium, and peritoneum [94], and in several human cancers, such as mesotheliomas and adenocarcinomas [95,96]. It has been shown that circulating mesothelin has a very high sensitivity for advanced stages of malignant pleural mesothelioma (MPM) [97]. Interestingly, in a study using mesothelioma markers in cytological specimens, it was concluded that calretinin was a better marker than mesothelin [98]. 

Cytokeratins and Thrombomodulin: Mesothelial cells typically express high levels of cytokeratins [99,100]. In a study by Cury and colleagues, an antibody to cytokeratin 5/6 antibody was used in combination with antibodies recognizing three others mesothelium-associated markers (thrombomodulin, calretinin, and CD44) to distinguish epithelioid pleural mesothelioma from metastatic adenocarcinoma. While each antibody stained reactive mesothelium, some of the antigens were also expressed in others cell types. The authors concluded that only calretinin, cytokeratin 5/6, and thrombomodulin show sufficient specificity for practical use [101]. In another study, thrombomodulin was used in combination with an antibody for HBME-1, a known mesothelial marker [102]. However, in their study, Kennedy and colleagues showed that HBME-1 is not sufficiently specific to be used for differential diagnosis of malignant mesothelioma as carcinoma metastatic to pleura are also positive for HBME-1. The authors conclude that thrombomodulin is sufficiently specific as to be a useful discriminator of the mesothelial nature of the mesothelioma [103].

Protein phosphatase inhibitor-1 (inhibitor-1 or I-1): Protein phosphatase inhibitor-1 (I-1) is an endogenous inhibitor of protein phosphatase-1 involved in signal transduction. Expression studies in embryonic and adult tissues have demonstrated that I-1 is expressed in the coelomic epithelium of the kidney, lung, liver, heart, intestine, and gonad, which has led to the conclusion that I-1 is a mesothelial marker [104].

Wilms’ tumor susceptibility gene 1 (WT1): As described in Section 3, the transcription factor WT1 is expressed in embryonic mesothelial structures and involved in the development of mesothelial-associated organs. In the adult, expression of WT1 is confined to the gonads, mesothelial tissues, and podocytes. Because of this, WT1 was suggested as a useful marker for the mesothelial cell lineage and a molecular tool in the diagnosis of cancers originating from this cell population [45]. The value of WT1 as a marker has, for instance, been demonstrated in a study by Ordonez, in which 60 epithelioid mesotheliomas and 50 lung carcinomas were analyzed with a panel of different immunohistochemical markers. In this study it was reported that 93% of the mesotheliomas were found to be positive for WT1, while none of the carcinomas showed WT1 reactivity, pointing to WT1 as a very good positive marker for mesothelioma [96]. 

microRNAs (miRNAs): miRNAs are small sequences of RNA that can regulate the expression of genes. Recent studies have shown that specific circulating miRNAs can be found in the serum of patients suffering from MPM. This set of miRNAs include miR-197-3p, miR-1281, and miR-32-3p [105,106]. This relatively recent insight into the presence of “disease-specific” miRNAs will not only help and refine the diagnosis of mesotheliomas, and specifically MPM, it might also open the door to the development of new therapies for these diseases [107]. 

In this section, we have provided a short list of markers used in the diagnosis of mesotheliomas. There are many more that could be added to this list. It is up to researcher and clinicians in the field to determine which markers (typically a combination of several positive and negative markers) should be used in any given situation. 

## 5. Pathological Conditions of Mesothelium-Derived Tissues

Serous membranes (or serosal membranes) consist of two layers of mesothelium between which serous fluid is secreted. This fluid is important for lubrication and protection of the coelomic organs. Serous effusion is characterized by an increased amount of fluid accumulating within the serosal cavity. It can affect all the pleural, pericardial, and peritoneal cavities [108]. It is usually associated with diseases, such as cancer or inflammation of the lining and organs of the respective cavities [109,110,111,112]. It is not uncommon in pleural effusion that the volume of the pleural fluid increases up to 1 to 1.5 L. The accumulation of liquids inside the cavities can induce pain and can affect the function of the organs. 

Inflammation is the most frequent disease condition of the serosal membranes and can have various origins. Among these are infectious diseases, such as tuberculosis [113] or Dressler syndrome, a form of pericarditis resulting from an immune response after damage to heart tissue [114]. Viral infections such as HIV promote bacterial infections and can also be the cause of inflammation of serosal membranes [115]. Cancer and inflammatory diseases, in particular autoimmune disorders such as systemic lupus erythematosus (SLE), kidney failure with uremia, radiation therapy, chemotherapy, and some medications may also lead to inflammation of the mesothelium-derived serosa [116,117]. Relatively new is the concept of inflammasomes. An inflammasome typically consists of several NOD-like receptors (NLRs), which are intracellular sensors of microbial motifs that can detect endogenous danger or stress signals (danger-associated molecular patterns, DAMPs) leading to inflammation [118]. The NLRP3 (NALP3 or cryopyrin) inflammasome has been associated with activation of the innate immune system in pathogenic particle-associated (e.g., asbestos and silica) inflammation [119,120,121] 

The mesothelium can also be affected by tumors. The most common is the metastatic spread of tumor cells from distant sites. However, primary tumors of the serosa can also affect the pleural, pericardial, and peritoneal serosa [122]. These primary cancers of the mesothelium-derived tissues are referred to as mesotheliomas. They are unfortunately typically detected at advanced stages. It can be difficult to differentiate these two malignant conditions as cases of metastatic invasion from mesothelioma have also been reported [122,123,124,125]. Compounds such as asbestos can cause chronic inflammation that could lead to the development of mesothelioma [126]. Below, we will discuss in more detail aspects of this devastating disease.

## 6. Malignant Pleural Mesothelioma and Its Etiologies

Malignant mesothelioma is a neoplasm that originates from mesothelial cells lining the body cavities including the pleura, pericardium, peritoneum, and tunica vaginalis. Approximately 70 to 80% of reported mesotheliomas develop in the pleural cavity and are known as Malignant Pleural Mesothelioma (MPM) [1]. The other 20% arise in the peritoneum, with the pericardium and the tunica of the testis and ovary being rarely affected [4]. MPM is considered a relatively rare type of cancer. The overall incidence rate of MPM is approximately 1 per 100,000 in the United States and 1 to 3 per 100,000 in the majority of European countries [2,127]. The rates for individuals with little or no occupational asbestos exposure is, however, significantly less (1 per 1,000,000) [128]. MPM is considered a major public health issue given the aggressiveness of the disease and the resistance to available treatments; on average, mesothelioma patients survive 12 to 21 months. In this section we will discuss some aspects of what is currently known about the etiologies leading to MPM. 

Asbestos and asbestos-like compounds: Asbestos exposure is implicated in ~80% of MPM cases. However, in the remaining 20% of cases there is no indication of asbestos exposure, suggesting that other etiologic factors including genetic susceptibility in certain individuals may be involved [129]. The link between MPM and exposure to asbestos was first demonstrated more than 50 years ago by an epidemiological study on a cohort of individuals working in asbestos mines in South Africa [130]. Asbestos are hydrated mineral silicates that contain a fibrous texture. Asbestos fibers are minerals with exceptional physical and chemical properties. They are fireproof, are remarkably resistant to various chemicals, and have a high mechanical tensile strength. These properties have allowed for the development of the use of asbestos fibers in many applications including the manufacturing of many industrial consumer products and in the construction of buildings. Exposure to asbestos fibers is therefore an occupational hazard, with the incidence of MPM among professionally exposed persons at 40 times higher than that observed in the general population [131]. While asbestos was banned from use in more than 50 countries, the mean latency period between asbestos exposure to the time of MPM diagnosis is in the range of 15 to 50 years [132,133,134]. In a pooled analysis of eight cohort studies, it was found that the median latency period is approximately 39 years [135]. The same study also noted that women tend to have a longer latency period compared to men [135]. Despite the cessation of asbestos use in many countries, a peak in the incidence of asbestos-related MPM is expected in the next decades due to the long latency between exposure to asbestos and the onset of the disease [1,136]. 

The carcinogenicity of asbestos fibers is related to their physical properties, in particular their size and diameter. There are two basic groups of asbestos: serpentine and amphibole. The risk to develop MPM appears highest when exposed to amphiboles [137]. Fibers of the amphibole family, especially crocidolite, have a clearly established carcinogenic role [138]. These fibers are long and fine (size greater than 5 μm and diameter less than 0.25 μm), and several studies have shown that long asbestos fibers (LAFs) have a greater carcinogenic effect than short asbestos fibers (SAFs) [139,140,141]. Inhaled asbestos fibers pass from the pulmonary alveoli to the pleural space [142], and the mechanism by which these fibers cause MPM has been well-described [143]. Any particle that enters the pleural space is normally eliminated to the nearest lymph nodes by the pleural fluid which performs a rapid turnover through the stomata—openings connecting the parietal pleura to the lymphatic system. While short fibers such as chrysotile can be disposed of, LAFs cannot pass through the stomata due to their size. These long fibers then accumulate around these openings at the level of the parietal pleura, thereby forming black spots. This retention of asbestos fibers in the parietal pleura then leads to biological events that lead to the onset of disease. The persistence of asbestos fibers, coupled with the inability of these fibers to dissolve or fragment into shorter fibers, can cause pleural irritation due to repeated damage to the mesothelial surface followed by proinflammatory tissue repair cycles [144,145]. 

Mesothelial cells can phagocytize asbestos fibers causing intracellular oxidation and DNA breaks [146,147,148,149]. It has also been shown that these fibers interfere with mitosis resulting in aneuploidy due to abnormal segregation of chromosomes during this process [150]. Finally, when pleural macrophages attempt to phagocytize the asbestos fibers, the size of the fibers prevents proper phagocytosis. This is causing so-called frustrated phagocytosis, which leads to the release of proinflammatory cytokines, free radicals, and reactive oxygen species [143]. This oxidative stress can indirectly participate in asbestos-induced genetic damage to the DNA in the mesothelial cells [151,152]. Upon contact with asbestos fibers, macrophages produce Tumor Necrosis Factor alpha (TNF-α), which is a key cytokine involved in inflammatory processes such as acute phase reaction, systemic inflammation, and chronic inflammation [153,154,155,156]. TNF-α binds to TNF-α Receptor 1 located on mesothelial cells, thereby activating NF-kB. Activation of the NF-κB pathway allows mesothelial cells with DNA damage as a result of exposure to asbestos fibers to survive and proliferate [157,158]. In addition, it has been shown that asbestos fibers can also cause necrosis of mesothelial cells resulting in the release of High Molecular Group Binding protein 1 (HMGB1), promoting an inflammatory response and the accumulation of TNF-α producing macrophages [159,160]. Asbestos fiber can also induce the MAPK/ERK cascade through autophosphorylation as well as through autophosphorylation of epidermal growth factor receptor (EGFR) [161]. TNF-α cytokine, NF-κB, and MAPK/ERK cascades have all been described in the inflammation process as well as in carcinogenesis, and are reported to be at least partially responsible for the switch from inflammation to cancer [162,163,164].

There are a few asbestos-like components that are also implicated in the onset of MPM, including erionite, nanoparticles, multiwalled, and single-walled carbon nanotubes (CNTs), silicon carbide (SiC) whiskers, and fluoro-edenite [165,166,167,168,169]. Erionite is a naturally occurring fibrous zeolite compound that has been recognized as a carcinogen involved in MPM for several years. A connection between erionite and MPM was reported in an animal study where an extremely high incidence of MPM (more than 90%) was found in mice following intraperitoneal injections of erionite [166]. A strong connection between erionite and MPM was also found in humans. In Turkey, populations exposed to erionite have very high rates of MPM [170]. A very interesting and important observation was reported for erionite-associated MPM in two separate villages in the region of Cappadocia in Turkey. While erionite was found in the homes of both villages, the populations of the two villages that were exposed to erionite showed a very different incidence of mesothelioma. In one village, half of the men died from mesothelioma, while in the other village only one case was reported [171]. The mapping of genetic trees in these populations suggested a genetic predisposition to developing MPM [171,172]. This hypothesis was challenged by others that suggested that the risk of MPM was due to indoor exposure to erionite, and that familial clustering might be coincidental, or, alternatively, that erionite might be contributing to MPM in genetically sensitive individuals [173]. Multiwalled carbon nanotubes morphologically resemble asbestos fibers including needle-like shape, high durability, and propensity to migrate to the pleura when inhaled [169]. Injection of long multiwalled carbon nanotubes into the peritoneal cavity of mice induced inflammation, suggesting that these nanotubes have asbestos-like pathogenicity [168]. This has made them a potential risk factor of MPM. Long multiwalled carbon nanotubes also caused granuloma formation in the pleura similar to long asbestos fibers. Thin and high crystallinity carbon nanotubes have been shown to pierce the mesothelial cell membrane, induce cytotoxicity in vitro, and initiate inflammation with subsequent transformation of mesothelial cells in vivo [167]. While no direct association between human exposure to carbon nanotubes and development of MPM have been reported, the fact that both erionite and multiwalled carbon nanoparticles share similarities with asbestos suggests that the morphology and size of the particles are of critical importance in the potential to access the pleural tissue and induce an inflammatory response that can lead to development of MPM.

Simian virus 40 (SV40): SV40 belongs to the family of Papovaviridae. It is a polyomavirus with double-stranded DNA of monkey origin. It has been thought to be transmitted to humans through SV40 contaminated polio vaccines between 1955 and 1963 [174]. The potential role of SV40 in the development of mesothelioma is controversial. Although many people were infected with SV40 by the contaminated polio vaccines, none of the studies conducted, could conclusively demonstrate the involvement of SV40 in the development of MPM. This is mainly due to the conflicting laboratories results and because the overall incidence of MPM was not consistent with the millions of previously SV40-contaminated polio vaccines [175]. Nonetheless, since the 1970s, DNA sequences or gene products of SV40 have been frequently detected in different types of human tumors, and particularly in mesothelioma [176,177]. The identification of SV40 sequences by PCR was observed in 60% of human mesotheliomas specimens and was later confirmed in a multicenter study in the United States [178,179]. However, in other studies conducted outside the USA, SV40 was not detected in MPM suggesting possible geographical variations in the use of previously SV40-contaminated polio vaccine or differences in detection methods [180,181].

Radiation: Exposure to ionizing radiation or sequelae following pleural infection (chronic inflammation) has also been reported to play a role in the subsequent occurrence of MPM [4,182]. Ionizing radiation is definitely carcinogenic in humans and it is therefore plausible that high levels of irradiations of the pleura can cause mesothelioma as has been reported in patients receiving therapeutic radiation for, for instance, Wilms’ tumor or lymphoma, and in patients who received the contrast medium Thorotrast, a suspension containing particles of the radioactive compound thorium dioxide [183,184]. The causal association was further supported by development of MPM in rat models injected with radioactive material and a review on epidemiological studies found a statistically significant increase in MPM risk especially with Thorotrast and radiation therapy [185,186]. Although there is no definitive causal link between radiation and MPM, a possible causal association is evident from the case reports and the plausible oncogenic actions of radiation.

## 7. Mesothelioma, DNA Damage, Cell Cycle Regulation, and Apoptosis

In the sections above, we have provided a short overview of the underlying causes of mesothelioma. In the last part of this review we will touch on a number of molecular and cellular mechanisms involved in the pathogenesis of the disease. Insight into how mesothelioma changes gene expression can be achieved by performing transcriptome studies. Such studies, performed on malignant mesothelioma cell lines, have shown significant changes in gene expression related to mesothelial cell transformation. Among the genes that show differential expression are genes involved in DNA repair, cell cycle, apoptosis, and other processes. Genes overexpressed in mesothelioma cell lines include for instance several cyclins (*CCND1*, *CCND2*), *CDK* phosphatase (*CDC25B*), *JNK1*, *NIK*, *TRAF2*, *PAK1*, *ERK5*, *JAGGED1* and the proto-oncogene *c-myc* [187,188]. 

Malignant mesothelioma has also been associated with recurrent deletions in specific chromosomal regions, including 1p, 3p, 6q, 9p, and 22q [189,190,191,192,193,194]. Here we will briefly describe the consequences of (some of) these deletions. 

The chromosomal deletions 9p21 and the 22q12 are well described as they lead to alterations of known signaling pathways crucial for maintaining cellular integrity through regulation of the cell cycle, cellular growth, and apoptosis during the establishment and progression of mesothelioma [195]. The coding region for *CDKN2A* is located in the 9p21 locus and its deletion leads to loss of p16^INK4A^, which is a critical cyclin-dependent kinase (CDK) inhibitor [196]. It also leads to the loss of p14^ARF^, a tumor suppressor and regulator of p53. The loss of p16^INK4A^ is associated with activation of the *CDK4* and *CDK6* which leads to inactivation of *pRB* and results in a feed-forward cycle as CDK4 becomes hyperphosphorylated leading to more activation. p14^ARF^ inhibits degradation of p53 through its interaction with murine double minute 2 protein (MDM2). Loss of p14^ARF^ leads to the activation of MDM2 resulting in a destabilization of p53 and eventually its degradation [197]. pRb is involved in the G1 to S checkpoint control and p53 protein plays a key role in apoptosis control and cellular senescence. The deletion of 9p21 in MM cells induces a cascade of events that eventually leads to the loss of G1 to S checkpoint control during cell cycle, weakens the ability to achieve apoptosis, and deregulates cell cycle control. The high frequency of deletion of the 9p21 locus observed in patients with mesothelioma is linked with increased malignancy.

The 22q12 chromosomal region contains the locus coding for the gene *NF2* which encodes the protein Merlin. Merlin is a critical component in the upstream regulation of the Hippo pathway and YAP1 [198]. Merlin is able to initiate the phosphorylation of proteins in the Hippo signaling pathway and eventually of the protein YAP when it is present in the cytoplasm. The phosphorylation of YAP prevents its entry to the nucleus and leads to its degradation. When Merlin is lost following a deletion of the 22q12 region, the cascade of phosphorylation in the Hippo pathways is lost as well, and YAP is able to enter the nucleus where it regulates genes involved in cell proliferation and death [199]. Studies have also revealed a loss of and/or mutations for several components of the Hippo pathway (e.g., *YAP*, *NF2*, *LATS2*, and *RASSF1*) in mesothelioma [200,201]. Modification of the Hippo pathway appears to be a key event in the carcinogenesis of mesothelioma. Loss of Merlin also has an effect on the PI3K and mTOR signaling pathways. Typically, Merlin inhibits the PI3K and mTOR pathways, preventing phosphorylation of AKT, which is involved in cell proliferation and resistance to apoptosis [202]. The loss of Merlin in malignant mesothelioma cells allows for an overexpression of the PI3K/Akt/mTOR pathway [203,204]. PTEN acts as a central regulator of AKT by inhibiting the formation of PIP3, a key activator of AKT [203]. A number of studies have shown a loss of PTEN in mesothelioma and an associated overexpression of the PI3K/Akt/mTOR pathway [205,206]. 

## 8. Conclusions

The mesothelium is an epithelium derived from the mesoderm and plays a crucial role in the development of organs that develop in the embryonic coelom, including the heart, lungs, and the intestines. Mesothelium-derived structures can be affected by a number of pathologies with the most serious pathological conditions being the primary cancers known as mesotheliomas. Various genes that are characteristically found in the developing and established mesothelial structures are also extremely valuable as markers in the diagnosis of mesotheliomas allowing to discriminate malignant mesotheliomas from, for instance, adenocarcinomas (e.g., *cytokeratin* and *WT1*). 
